# Effect of Normalization Methods on Accuracy of Estimating Low- and High-Molecular Weight PAHs Distribution in the Soils of a Coking Plant

**DOI:** 10.3390/ijerph192315470

**Published:** 2022-11-22

**Authors:** Yumin Yuan, Kai Yang, Lirong Cheng, Yijuan Bai, Yingying Wang, Ying Hou, Aizhong Ding

**Affiliations:** College of Water Sciences, Beijing Normal University, Beijing 100875, China

**Keywords:** data transformation, hotspots, uncertainty analysis, contaminated site, polycyclic aromatic hydrocarbons

## Abstract

Mapping spatial distribution of soil contaminants at contaminated sites is the basis of risk assessment. Hotspots can cause strongly skewed distribution of the raw contaminant concentrations in soil, and consequently can require suitable normalization prior to interpolation. In this study, three normalization methods including normal score, Johnson, and Box-Cox transformation were performed on the concentrations of two low-molecular weight (LMW) PAHs (i.e., acenaphthene (Ace) and naphthalene (Nap)) and two high-molecular weight (HMW) PAHs (i.e., benzo(a)pyrene (BaP) and benzo(b)fluoranthene (BbF)) in soils of a typical coking plant in North China. The estimating accuracy of soil LMW and HMW PAHs distribution using ordinary kriging with different normalization methods was compared. The results showed that all transformed data passed the Kolmogorov-Smirnov test, indicating that all three data transformation methods achieved normality of raw data. Compared to Box-Cox-ordinary kriging, normal score-, and Johnson-ordinary kriging had higher estimating accuracy of the four soil PAHs distribution. In cross-validation, smaller root-mean-square error (RMSE) and mean error (ME) values were observed for normal score-ordinary kriging for both LMW and HMW PAHs compared to Johnson- and Box-Cox-ordinary kriging. Thus, normal score transformation is suitable for alleviating the impact of hotspots on estimating accuracy of the four selected soil PAHs distribution at this coking plant. The findings can provide insights into reducing uncertainty in spatial interpolation at PAHs-contaminated sites.

## 1. Introduction

Soil contamination with polycyclic aromatic hydrocarbons (PAHs) generated from fossil fuel and biomass combustion is of serious global environmental concern due to the potential carcinogenicity and mutagenicity of PAHs [1]. PAHs in contaminated soils can pose a significant risk to human and ecological health via food chains and direct exposure pathways (e.g., inhalation, ingestion and dermal contact) [2]. Hazardous levels of PAHs in soils of coking plants have been frequently reported and drawn great attention of authorities and scientists [3,4]. Previous investigations revealed that concentrations of PAHs in the topsoils of coking plants may vary significantly depending on the distance from the production area [5], suggesting a spatially varying relationship between soil PAHs distribution and land use patterns at a coking plant. Moreover, due to the intrinsic physicochemical characteristics of PAHs, low-molecular weight (LMW) PAHs, which have relatively higher water solubility, are harder to be absorbed by soil organic matter (SOM) and easier to migrate in the soil compared to high-molecular weight (HMW) PAHs [6,7]. For example, the solubility of naphthalene (Nap), acenaphthene (Ace), benzo(a)pyrene (BaP), and benzo(b)fluoranthene (BbF) in water (25 °C) was 30.2, 3.9, 0.014, and 0.008 mg/L, respectively [8]. Biodegradation efficiency of soil PAHs with different numbers of rings has also been demonstrated to be significantly different, with HMW PAHs being reluctant for the oxidative degradation process [6]. The contrasting migration and transformation behaviors of LMW and HMW PAHs along with the dominant effect of production activities on soil PAHs distribution inevitably introduce non-negligible uncertainty in the investigation of PAHs-contaminated sites. Therefore, it is necessary to improve interpolation techniques for higher estimating accuracy of distribution of soil PAHs with different ring numbers at coking plant-contaminated sites.

In recent years, geostatistical techniques have been increasingly employed in the description and prediction of spatial variability of environmental parameters [9,10]. Ordinary kriging is one of the most preferred stochastic spatial interpolation methods [11]. It is known as the best linear unbiased predictor since it assumes that the mean value of the estimation error is equal to zero, therefore minimizing the variance of the estimation error [12]. Ordinary kriging has been widely used to interpolate spatial variability of soil contaminants at contaminated sites [13]. However, high concentration outliers often occur and cause non-normal distribution of contaminants at industrial contaminated sites (e.g., coking plants, mining and smelting sites, battery recycling, and manufacturing facilities where production activities are carried out within a defined zone) [14]. Accurate predictions are usually complicated by the presence of censored data (below the detection limit) and highly skewed raw data [15]. As a result, considerable differences often exist in the sample variogram from its regional counterpart, and geostatistical interpolation would be hindered. Problems caused by non-normality and skewness of raw data can be alleviated by correcting the skewness using appropriate data transformation methods [16]. Natural logarithmic, normal score, and Box-Cox transformation are most commonly applied to solve the non-normality and to reduce the effect of outliers on geostatistical analyses [17]. For example, Liu et al. [5] demonstrated that Johnson transformation produced more robust variograms than normal score and Box-Cox transformation for estimating severely skewed soil BbF concentrations at a coking plant-contaminated site.

To accurately define the pollution and remediation boundary of soil PAHs at a contaminated site is of great importance for risk assessment and establishment of effective remedial management. Until now, few studies have compared the influence of different data transformation methods on the robustness of normalization of soil LMW and HMW PAHs concentrations at contaminated sites. Thus, we hypothesized that (1) the smoothing effect induced by different transformed data on soil PAHs concentrations at hotspots would affect the accuracy of interpolation of soil PAHs distribution at the industrially contaminated site to different extents, and (2) the distribution patterns of soil LMW and HMW PAHs as affected by hotspots would be different due to their different migration behaviors. Consequently, the selection of normalization method would depend on soil PAH type. The findings can explore appropriate normalization methods for interpolating spatial distribution of soil PAHs with different ring numbers and migration abilities. The effort can improve the accuracy of contaminated site survey and create a reliable basis for risk management.

In this study, two LMW PAHs (i.e., Ace and Nap) and two HMW PAHs (i.e., BaP and BbF) were selected as soil contaminants of environmental concern at a typical coking plant according to the previous site investigation. Nap is one of the most important volatile PAHs [18], and it has been recently found to be the most abundant urinary PAH among the non-smoking US population [19]. Ace is a typical three-ring PAH. The migration of both Ace and Nap can be enhanced in an acidic soil environment [20]. Whereas, BaP and BbF are listed by International Agency for Research on Cancer (IARC) as Group 1 and Group 2B carcinogens, respectively, and tend to accumulate in topsoil of contaminated sites. Three data transformation methods including normal score, Johnson, and Box-Cox were employed to normalize the concentration data of selected soil PAHs. The aims of this study were to (1) compare the variograms of ordinary kriging of transformed data using different normalization methods, and (2) determine appropriate normalization method for interpolating distribution of soil PAHs with different rings at the coking plant. The findings can improve the reliable basis for risk assessment and accuracy for the determination of remediation boundary at PAHs-contaminated sites.

## 2. Materials and Methods

### 2.1. Site Description and Sampling Procedure

The historical coking plant was located in North China, with an operating history of more than 40 years. Due to the lack of technical capacity and pollution control in the early stage, the emission of carcinogenic pollutants resulted in severe damage to the site and surrounding environment. Soil PAHs were mainly sourced from leakages and spills during the process of gas purification and chemical production, storage, and transportation. In the northern part (with an area of approx. 1.5 km^2^) of the coking plant, a total of 60 soil samples were collected by Geoprobe 6620 DT using systematic grid sampling method at a depth of 0–50 cm. An aliquot of soil sample was taken from each sampling tube, fully packed in the sampling bottle and wellsealed at the sampling site. All soil samples were stored below 4 °C and analyzed for PAHs concentrations within one week.

### 2.2. Analytical Procedures

A total of 10 g of each soil sample was weighed and mixed with acetone/dichloromethane (1:1, *v*/*v*) as solvent. The sample was extracted using ASE-300 (Dionex, Sunnyvale, CA, USA) with 30 mL dichloromethane/n-hexane (2:1, *v*/*v*) as stated by Grimalt et al. [21]. The concentrated extracts of PAHs were analyzed using a gas chromatography-mass spectrometry (GC-MS) (Agilent, 6890N GC, 5975B MS detector, Santa Clara, CA, USA) equipped with a spitless injector, HP-5MS capillary column (30 m, 0.25-mm inner diameter × 0.25-mm film thickness, Agilent, USA). For quality control, matrix spike, duplicate, and laboratory blank were analyzed. All quality control samples were run with every 20 samples. The relative percentage difference (RPD) of duplicate samples was below 20%. The recoveries of the 16 individual PAHs from the matrix spike samples were all within the quality control ranges (e.g., 82–108% for Ace, 70–94% for Nap, 78–118% for BaP, and 85–113% for BbF).

### 2.3. Geostatistical Normalization

#### 2.3.1. Box-Cox Transformation

Box-Cox transformation is one of the widely used normalization methods [22,23], and the formulation is given by (1):

(1) $$y=\{\begin{array}{c} \frac{x^{\lambda \;}-1}{\lambda }\\ \mathrm{In}\left(x\right),\;\;\lambda =0 \end{array},\;\lambda \;\neq 0$$

where *y* is the value of transformation, *x* is the value to be transformed, and *λ* is based on the transformed values (*y_1_, y_2_, …, y_n_*) with an assumption of normal distribution. When *λ* = 0, the transformation is a logarithmic transformation.

#### 2.3.2. Normal Score Transformation

Zhang et al. [24] reported the function of normal score transformation in spatial analysis, where the raw data were ranked in ascending order and matched their ranks to equivalent ranks produced in the normal distribution. It is an efficient tool to transform the non-normality and skewed distribution of raw data to a nearly symmetrical distribution.

#### 2.3.3. Johnson Transformation

Johnson transformation introduced three kinds of distribution curve groups with reference to random variables, which can easily alleviate the non-normality and skewness of the raw data [25,26]. It can use different distribution curves for normal transformation of variables with different characteristics. There are three types of Johnson transformations: SB, SL, and SU, representing abounded, lognormal, and unbounded distributions, respectively. Johnson transformation is expressed as:

(2) $$Z=\gamma +\delta f\left[\frac{X-\xi }{\lambda }\right]$$

where Z is the standard normal distribution variable, *X* is the non-normal distribution variable, the parameters γ and δ control the shape of *X* distribution, ξ  is the position factor, and λ is the scale factor. Further details about these parameters can be found in previous publications [27,28].

### 2.4. Spatial Interpolation

#### 2.4.1. Kriging Methods

Kriging [29,30] is regarded as an optimal method of spatial prediction, which weighs the surrounding measured points to calculate a prediction for an unknown location. There are several types of kriging including simple kriging, universal kriging, cokriging, etc. Ordinary kriging is one of the most commonly applied methods [31]. Before kriging, the spatial variation of transformed variables and input parameters for kriging were modeled with the aid of semivariograms, where the weights of ordinary kriging were derived from the kriging equations using a semivariance function [29]. The chosen model was fitted by the weighted least squares through the points in the graph of semivariance so that the weighted squared difference between each point and the line was as small as possible [30]. The semivariances are calculated using the following equation:

(3) $$\gamma \left(h\right)=\frac{1}{2N\left(h\right)}\sum_{i=1}^{N\left(h\right)}{\left[z\left(x_{i}\right)-z\left(x_{i}+h\right)\right]}^{2}$$

where $z\left(x_{i}\right)$ is the measured value at the location of $x_{i}$, *h* is the lag distance and *N*(*h*) represents the number of samples at lag *h* apart, γ(h) refers to the semivariance value at distance interval *h*. For semivariogram variogram, different values of *h* can produce a series of γ(h) values. This is then generally fitted with a theoretical model such as spherical, Gaussian, linear, exponential models, etc., with nugget (*C*_0_), range and sill (*C*) being three important parameters.

Ordinary kriging is regarded as an optimal spatial interpolation method, which is a type of weighted moving average [32]. The formula of ordinary kriging interpolation is as follows:

(4) $$\hat{z}\left(x_{0}\right)=\sum_{i=1}^{n}\lambda_{i}z\left(x_{i}\right)$$

where $\hat{z}\left(x_{0}\right)$ is the value to be estimated at the location of $x_{0}$, $z\left(x_{i}\right)$ is the known value at the sampling site and *i* is the number of sites within the search neighborhood used for the estimation. The number *n* is based on the size of the moving window and defined by the user [33].

#### 2.4.2. Evaluation of Interpolation Method

Due to limited number of samples, cross-validation was conducted to evaluate the performance of ordinary kriging with different data transformation methods [34]. The models were judged by comparing the mean error (ME), root-mean-square error (RMSE), average standard error (ASE), and root-mean-square standardized error (RMSSE) calculated from the measured and interpolated values at each sample site. To find out which model is optimal in predicting values, ME, RMSE, ASE, and RMSSE were calculated using the following equations (Equations (5)–(8)):

(5) $$\mathrm{ME}=\frac{1}{n}\sum_{i=1}^{n}\left[Z\left(x_{i}\right)-Z^{*}\;\left(x_{i}\right)\right]$$

(6) $$\mathrm{RMSE}=\sqrt{\frac{1}{n}\sum_{i=1}^{n}{\left[Z\left(x_{i}-Z^{*}\left(x_{i}\right)\right)\right]}^{2}}$$

(7) $$\mathrm{ASE}=\sqrt{\frac{1}{n}\sum_{i=1}^{n}\sigma^{2}\left(x_{i}\right)}$$

(8) $$\mathrm{RMSSE}=\sqrt{\frac{1}{n}\sum_{i=1}^{n}{\left[\left(Z\left(x_{i}\right)-Z^{*}\left(\left(x_{i}\right)\right)∕\hat{\sigma }\left(x_{i}\right)\right)\right]}^{2}}$$

where $Z\left(x_{i}\right)$ is the observed value of *Z* at location $x_{i}$, $Z^{*}\;\left(x_{i}\right)$ is the interpolated value at the same location, and *n* is the sample size. ME refers a measure of bias, RMSE provides a measure of accuracy, ASE is mean of prediction standard error, and RMSSE should be close to 1 if the prediction standard errors are valid. Smaller ME and RMSE values indicate a more accurate interpolation.

### 2.5. Software

The tests for normality and data transformation were carried out in SPSS 21.0 and Minitab 17.0. The geostatistical analyses were performed in GS+ 9.0. All maps were produced using ArcGIS 10.2 with Geostatistical Analyst extension.

## 3. Results

### 3.1. Soil Ace, Nap, BaP, and BbF Concentrations

**Figure 1:**
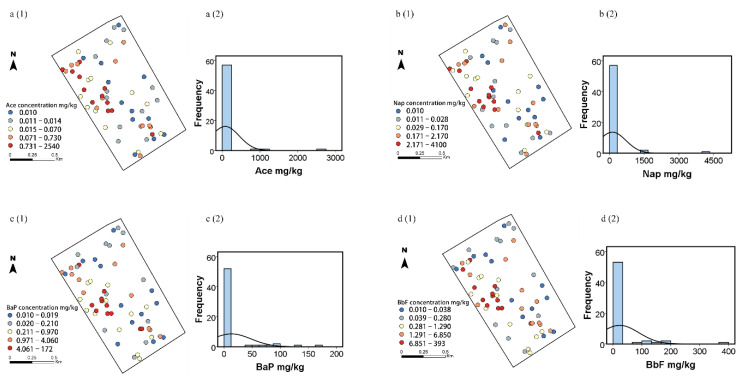
*Left*: Blot plots of soil Ace (**a1**), Nap (**b1**), BaP (**c1**), and BbF (**d1**) concentrations. *Right*: Histograms of soil Ace (**a2**), Nap (**b2**), BaP (**c2**), and BbF (**d2**) concentrations.

Concentrations of the selected PAHs in the soils are summarized in Table 1. All four PAHs concentrations exhibited a wide range of variation of several magnitudes, e.g., Ace varied between 0.01 and 2540 mg/kg. The mean Nap, BaP, and BbF concentrations exceeded the risk screening values (RSVs) for soil Nap (70 mg/kg), BaP (1.5 mg/kg), and BbF (15 mg/kg) in development land in China (GB36600-2018), being 2-, 8-, and 1.5-times higher than the corresponding RSVs, respectively. All mean concentrations of the four PAHs were conspicuously higher than the corresponding median concentrations, indicating a positively skewed distribution. Moreover, blot plots of soil Ace, Nap, BaP, and BbF concentrations across the study area are illustrated in Figure 1(a1,b1,c1,d1). The high concentrations of the four PAHs shown as hotspots were found in the western side, some relatively low values were noted in the eastern and northern sides, resulting in a significant skewness in the raw data. The positive skewness features with a long tail extending towards the high-value side are observed in the histograms (Figure 1(a2,b2,c2,d2)). Both histograms and frequency distributions of the four PAHs showed that the raw data had a non-normal distribution and high-value outliers, which agrees with the high skewness and kurtosis values and Kolmogorov-Smirnov (K-S) *p* values of the four PAHs.

To achieve stable variograms and kriging results, data transformation must be carried out for the raw data to limit the effects of skewness and outliers, and to solve the non-normality problem. The results of data transformation using Johnson, normal score, and Box-Cox transformation methods are shown in Table 2. All transformed data passed the K-S test, indicating that all three data transformation methods alleviated the heavily skewed raw data (Table 2). Compared with Johnson and Box-Cox transformation, normal score transformation significantly decreased the skewness and kurtosis of the four PAHs, pushing them towards symmetric or near normal distribution.

### 3.2. Spatial Structure of Soil Ace, Nap, BaP, and BbF Concentrations

**Figure 2:**
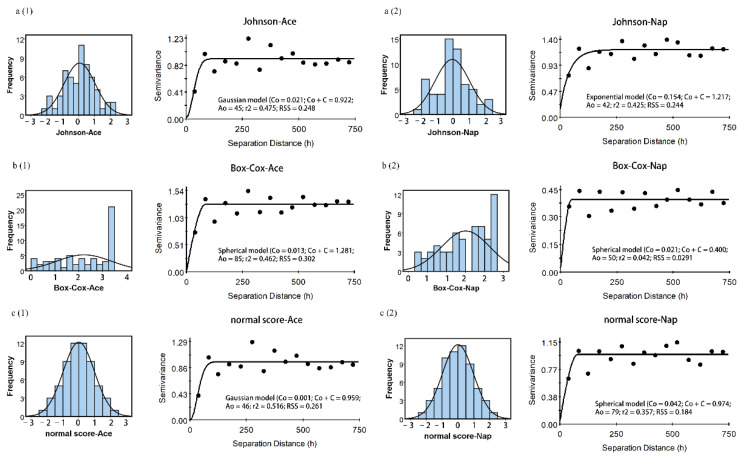
*Left*: Histograms of the transformed soil Ace (**a1**,**b1**,**c1**) and Nap (**a2**,**b2**,**c2**) concentrations: (a) Johnson transformed data; (b) Box-Cox transformed data; (c) normal score transformed data. *Right*: Experimental semivariograms and fitted parameters corresponding to the transformed data.

**Figure 3:**
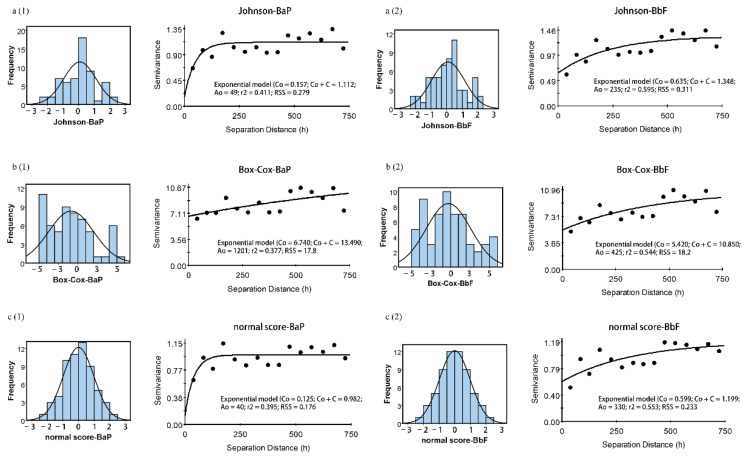
*Left*: Histograms of the transformed soil BaP (**a1**,**b1**,**c1**) and BbF (**a2**,**b2**,**c2**) concentrations: (a) Johnson transformed data; (b) Box-Cox transformed data; (c) normal score transformed data. *Right*: Experimental semivariograms and fitted parameters corresponding to the transformed data.

The semivariograms of Johnson, normal score, and Box-Cox transformed data were calculated to describe the spatial variation of the four PAHs concentrations (Figure 2 and Figure 3). The exponential model best characterized the structure of semivariograms of BaP and BbF while the spherical, exponential, and Gaussian models for Ace and Nap. The nugget values of all semivariograms were small (Table 3), except for Box-Cox transformed BaP and Nap concentrations, indicating that the sampling density can reveal the spatial structures more clearly. Among the three models, all nugget/sill ratios of Ace and Nap were lower than 25%, showing that LMW PAHs concentrations in this sampling scale had a strong spatial correlation. For BaP and BbF, the ratio values lower than 25% and higher than 75% corresponded to a weak and strong spatial correlation, respectively. This spatial dependence was found in the normal score and Johnson transformed data, which were probably attributed to the distribution of patches of contaminated soils. The nugget/sill ratios of BaP and BbF were 49.9% for Box-Cox transformed data, indicating that these variables had a moderate spatial dependency. It is worth noting that the semivariogram range of Box-Cox transformed BaP and Nap concentrations were 10-times greater than that of normal score- and Johnson transformed data and showed a higher nugget/sill ratio, illustrating its weak spatial structure as compared to its correspondence. Moreover, the relatively high regression coefficient (*r*^2^) and small residual sum of squares (RSS) of Johnson and normal score transformed data suggested that they had a high degree of confidence.

### 3.3. Spatial Distribution of Soil Ace, Nap, BaP, and BbF Concentrations

**Figure 4:**
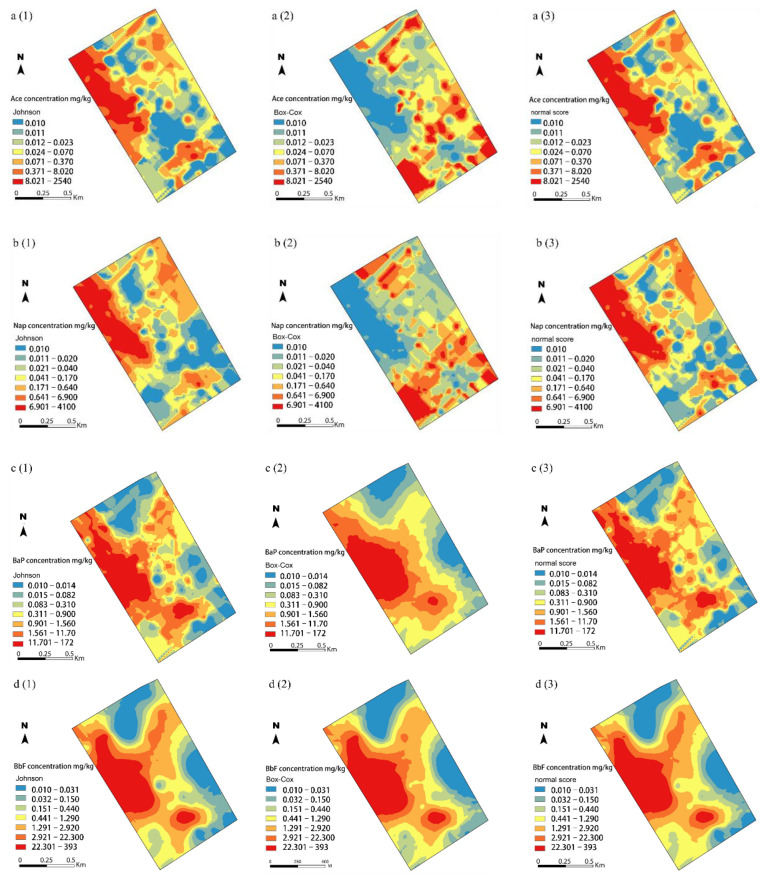
Spatial interpolation of transformed soil Ace (**a1**–**a3**), Nap (**b1**–**b3**), BaP (**c1**–**c3**), and BbF (**d1**–**d3**) concentrations: (1) Johnson transformed data; (2) Box-Cox transformed data; (3) normal score transformed data.

For spatial interpolation, the Johnson, normal score, and Box-Cox transformed data were applied to ordinary kriging based on the semivariogram models (Figure 4). For Ace (Figure 4(a1–a3)) and Nap (Figure 4(b1–b3)), similar spatial patterns were noted for the normal score and Johnson transformed data, with high concentration patterns located in the central-western area and several small hotspots scatted in the northern and southern areas. Box-Cox-ordinary kriging for Ace and Nap failed to predict the high concentration patterns in the central-western area (Figure 4(a2,b2)), with the high value patterns of Ace and Nap concentrations present in clearly opposite directions compared to those of Johnson- and normal score-ordinary kriging. In Figure 4(c1–c3,d1–d3), the BaP and BbF concentrations showed similar spatial patterns among the three data transformation methods: areas with high values mainly located in the central-western and southern areas, whereas areas with low values scattered around the study area, indicating that they may have been affected by the similar source in the study area. Despite the four PAHs concentrations exhibited a similar spatial distribution, there was a slight difference in the extent of contaminated areas based on the three data transformation methods. It can be clearly observed that normal score-ordinary kriging was effective in identification of a few hotspots of the four PAHs in the northeastern and southern areas while Box-Cox- and Johnson-ordinary kriging failed to reflect the fact. Considering the smoothing effect of all three data transformation methods, the gradients in prediction maps of the four PAHs were smaller for normal score- and Johnson-ordinary kriging.

### 3.4. Interpolation Accuracy

To compare the performance and accuracy of the spatial interpolation, the ME, RMSE, ASE, and RMSSE were determined for ordinary kriging combined with different data transformation methods (Table 4). The ASE and RMSSE values did differ greatly for the three methods, indicating that the spatial variability in prediction was overestimated. The normal score- and Johnson-ordinary kriging had relatively low RMSE and ME values for the four PAHs. The Box-Cox-ordinary kriging had the largest RMSE values for Ace, BaP, and BbF, and smallest RMSE values for Nap. In terms of accuracy, the results showed that normal score-ordinary kriging brought more robust estimation for the four PAHs compared to Box-Cox- and Johnson-ordinary kriging.

## 4. Discussion

The present study showed that each of the three data transformation methods had its unique advantages and produced slightly different prediction maps. For a clearer comparison, histograms were created for the four soil PAHs (Figure 2 and Figure 3). The normal score transformed data exhibited a normal distribution as reflected by the symmetric bell-shaped histogram, whereas the other two datasets followed asymmetric distribution. This is due to the fact that normal score transformation converted both high and low values to be evenly distributed by ranking them in order [35]. A clearly multi-peak feature of the four PAHs was shown for Johnson and Box-Cox transformation, indicating that the two methods over-transformed the variables, changing their skewness from positive to negative values [35]. However, it is acknowledged that the regression coefficient (*r*^2^) values for all three methods were relatively low (Table 3), which could be ascribed to the mixture of populations, detection limit problems, and size of samples [35]. Zhang et al. [35] demonstrated that factors such as geology, soil type, outliers, detection limits, and sample size could affect the results of statistical tests. Similarly, Shamsudduha [36] and Gong et al. [37] also highlighted that low sample densities led to lower predication accuracy due to varying biogeochemical and geological processes in the region. Meanwhile, there were some large and small values of the four PAHs concentrations adjacent to each other (Figure 1), implying the presence of possible spatial outliers which could contribute to the large nugget effect. Despite relatively low regression coefficient (*r*^2^) values of the three data transformation methods, the normal score transformation was efficient in normalizing the soil PAHs concentrations and reducing the skewness, which ensured that the data were stationary as required for ordinary kriging.

Pollutant leakages during tar processing, refining and gas purification, storage, and transport are usually the main causes of strong heterogeneity of soil contaminants at a coking plant [5]. Meanwhile, due to their special characteristics, PAHs with different ring numbers may possess different accumulation and migration behaviors in soil and cause different levels of soil contamination [7]. Overall, the concentrations of soil Ace, Nap, BaP, and BbF exhibited a similar spatial distribution pattern across the study area (Figure 4). However, there were slight differences in the spatial outliers between the three data transformation methods. As shown in Figure 4(a1–a3,b1–b3), most sampling sites with Ace and Nap over 900 mg/kg were found in the northwestern and southeastern areas for normal score- and Johnson-ordinary kriging, while this spatial pattern of elevated LMW PAHs was not available on the Box-Cox-ordinary kriging maps. The high concentration patterns of Ace and Nap showed spatial association with coal combustion and heavy oil, which were emitted by tar production and gas purification during the coke production process [38]. Moreover, the prediction maps of normal score- and Johnson-ordinary kriging corresponded to the distribution of pollutant production process workshops and exhibited more robust variograms of soil Ace and Nap concentrations in the northeastern and southern areas. Compared to Box-Cox-ordinary kriging (Table 4), normal score- and Johnson-ordinary kriging were more accurate in predicting spatial distribution of LMW PAHs concentrations in the soils. Despite of the smoothing effect of kriging, the gradients in the prediction maps of soil BaP and BbF concentrations were detailed and smaller in normal score-ordinary kriging than Johnson- and Box-Cox-ordinary kriging (Figure 4(c1–c3,d1–d3)). A previous study demonstrated that Johnson transformation was an optimal normalization method to improve accuracy of spatial interpolation of soil BbF at a coking plant [5]. Furthermore, slightly high value patterns of BaP and BbF concentrations were observed in the northeastern and southern areas, implying the effects of historical pollution from leached coal piles and coal loading. HMW PAHs have strong accumulation characteristics and low vapor pressure and are expected to persist or be absorbed strongly compared to LMW PAHs [7]. Despite the mapping performance of the three methods were similar, the smoothing effect of high-value outliers were obviously decreased in the prediction maps created using normal score-ordinary kriging, resulting in an increase in the estimating accuracy. Besides, normal score-ordinary kriging showed the lowest value of RMSE and relatively high regression coefficient (*r*^2^) value. These results demonstrated that normal score transformation performed better than the other two methods in estimating the spatial variation characteristic of soil PAHs with different ring numbers at the same sampling density.

Accurate prediction of spatial distribution of soil PAHs concentrations at a contaminated site is of great importance for risk assessment and establishment of effective remedial management [38]. Previous studies on the accuracy and uncertainty analysis mainly focused on the accuracy of prediction by using different interpolation methods. Despite data transformation is known to reduce skewness in order to obtain a near-normal distribution, different normalization methods may affect the accuracy of interpolation to different extents. In this study, a long tail toward high concentrations was observed for the four PAHs, indicating large difference of enrichment and strong heterogeneous characteristic of them. All three data transformation methods not only improved the non-normality distribution of the four PAHs concentrations, but also caused a smoothing effect inherited from ordinary kriging on hotspots. Both Johnson- and normal score-ordinary kriging exhibited a centralization effect on the four PAHs concentrations, with some of the high peak values underestimated and low values overestimated. Whereas, Box-Cox-ordinary kriging caused a strong smoothing effect and greater error in their predictions at low concentrations, indicating that Box-Cox over-transformed the data, especially for LMW PAHs. Moreover, compared to normal score transformed results, the spatial pattern changes were smoother in the case of Johnson transformed data.

## 5. Conclusions

The raw soil Ace, Nap, BaP, and BbF concentrations at the northern part of the historical coking plant were strongly positively skewed with several high peak values. After normal score, Johnson, and Box-Cox transformation, all transformed data passed the K-S test, indicating that all three data transformation methods achieved normality of raw data. Compared to Box-Cox-ordinary kriging, normal score- and Johnson-ordinary kriging had higher estimating accuracy of the four soil PAHs distribution. Based on the spatial distribution of soil Ace, Nap, BaP, and BbF concentrations and cross-validation, the smoothing effect of hotspots in neighboring area on interpolation could be alleviated sharply by normal score-ordinary kriging, and thus providing more accurate predictions for the areas around the hotspots. This study demonstrated that normal score transformation was suitable for improving the estimating accuracy of soil LMW and HMW PAHs despite their contrasting migration behaviors at the coking plant-contaminated site.

## Figures and Tables

**Table 1:** Summarized statistics of the raw soil PAHs concentrations (Unit: mg/kg).

PAH	Minimum	Maximum	Mean	Median	Skewness	Kurtosis	CV	SD	K-S Test	RSV ^a^
Ace	0.01	2540	80.86	0.03	5.64	34.42	4.59	371.45	Non-normal	--
Nap	0.01	4100	122.84	0.05	5.95	38.33	4.75	583.49	Non-normal	70
BaP	0.01	172	13.32	0.33	3.05	9.25	2.63	34.98	Non-normal	1.5
BbF	0.01	393	23.10	0.61	3.89	17.36	2.86	66.06	Non-normal	15

SD standard deviation, CV coefficient of variation, K-S test Kolmogorov-Smirnov test at the 0.05 level. ^a^ RSV risk screening value, sourced from the national standard Soil environmental quality—Risk control standard for soil contamination of development land (GB36600-2018).

**Table 2:** Summarized statistics of the transformed soil PAHs concentrations.

PAH	Transformation	Skewness	Kurtosis	*p*	K-S Test
Ace	Johnson	−0.034	−0.144	0.997	Normal
	Box-Cox	−0.461	−1.264	0.038	Normal
	Normal score	0.005	−0.242	0.010	Normal
Nap	Johnson	0.061	−0.126	0.743	Normal
	Box-Cox	−0.471	−1.052	0.167	Normal
	Normal score	0.002	−0.233	0.393	Normal
BaP	Johnson	−0.12	−0.168	0.880	Normal
	Box-Cox	0.493	−0.614	0.620	Normal
	Normal score	0.001	−0.233	0.999	Normal
BbF	Johnson	0.006	−0.347	0.972	Normal
	Box-Cox	0.438	−0.559	0.923	Normal
	Normal score	0.002	−0.236	0.999	Normal

**Table 3:** Modelled semivariogram parameters of soil PAHs concentrations.

PAH	Model	Nugget (*C*_0_)	Sill (*C*_0_ + *C*)	Proportion [*C*_0_/(*C*_0_ + *C*)]	Range (*A*_0_)	*r* ^2^	Residual SS
J-Ace	Gaussian	0.021	0.922	2.28	45	0.475	0.248
J-Nap	Exponential	0.154	1.217	12.65	42	0.425	0.244
J-BaP	Exponential	0.157	1.112	14.12	49	0.411	0.279
J-BbF	Exponential	0.635	1.348	47.11	235	0.596	0.311
B-Ace	Spherical	0.013	1.281	1.01	85	0.462	0.302
B-Nap	Spherical	0.021	0.400	5.25	50	0.042	0.029
B-BaP	Exponential	6.740	13.490	49.96	1201	0.377	17.800
B-BbF	Exponential	5.420	10.850	49.95	425	0.544	18.100
N-Ace	Gaussian	0.001	0.959	0.10	46	0.516	0.261
N-Nap	Spherical	0.042	0.974	4.31	79	0.357	0.184
N-BaP	Exponential	0.125	0.982	12.73	40	0.395	0.176
N-BbF	Exponential	0.599	1.199	49.96	330	0.553	0.233

J: Johnson transformation; B: Box-Cox transformation; N: Normal score transformation.

**Table 4:** Cross-validation indices for different data transformation methods.

PAH	Prediction Model	ME	RMSE	ASE	RMSSE
Ace	Normal score-ordinary kriging	0.003	0.863	1.026	0.838
	Johnson-ordinary kriging	0.002	0.855	1.019	0.836
	Box-Cox-ordinary kriging	−0.024	1.007	−0.005	0.886
Nap	Normal score-ordinary kriging	0.014	0.951	1.032	0.921
	Johnson-ordinary kriging	−0.032	1.036	1.247	0.833
	Box-Cox-ordinary kriging	0.009	0.589	0.687	0.856
BaP	Normal score-ordinary kriging	−0.013	0.908	1.121	0.815
	Johnson-ordinary kriging	−0.006	0.970	1.196	0.817
	Box-Cox-ordinary kriging	0.100	2.647	3.332	0.811
BbF	Normal score-ordinary kriging	0.046	0.904	1.212	0.758
	Johnson-ordinary kriging	0.057	0.980	1.336	0.744
	Box-Cox-ordinary kriging	0.158	2.591	3.500	0.757

## Data Availability

The data that support the findings of this study are available from the corresponding and lead authors, upon reasonable request.
